# Tetra­ammineplatinum(II) dichloride ammonia tetra­solvate

**DOI:** 10.1107/S1600536814012343

**Published:** 2014-06-07

**Authors:** Tobias Grassl, Nikolaus Korber

**Affiliations:** a Institut für Anorganische Chemie, Universität Regensburg, Universitätsstrasse 31, 93053 Regensburg, Germany

## Abstract

The title compound, [Pt(NH_3_)_4_]Cl_2_·4NH_3_, was crystallized in liquid ammonia from the salt PtCl_2_. The platinum cation is coordinated by four ammonia mol­ecules, forming a square-planar complex. The chloride anions are surrounded by nine ammonia mol­ecules, either bound within the platinum complex or solvent mol­ecules. The solvent ammonia mol­ecules are packed in such a way that an extended network of N—H⋯N and N—H⋯Cl hydrogen bonds is formed. The structure is isotypic with [Pd(NH_3_)_4_]Cl_2_·4NH_3_ [Grassl & Korber (2014). *Acta Cryst.* E**70**, i32].

## Related literature   

For weak inter­molecular inter­actions such as hydrogen bonds and their application in crystal engineering, see: Desiraju (2002 ▶, 2007 ▶); Steiner (2002 ▶). For the structure of Magnus salt and tetra­amminplatinous salts, see: Atoji *et al.* (1957 ▶); Cox (1932 ▶); Smolentsev *et al.* (2010 ▶). The Pd analogue is described by Grassl & Korber (2014 ▶).

## Experimental   

### 

#### Crystal data  

[Pt(NH_3_)_4_]Cl_2_·4NH_3_
*M* *_r_* = 402.25
Monoclinic, 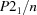

*a* = 7.6641 (2) Å
*b* = 10.1601 (3) Å
*c* = 8.7797 (2) Å
β = 100.975 (3)°
*V* = 671.15 (3) Å^3^
*Z* = 2
Mo *K*α radiation
μ = 10.83 mm^−1^
*T* = 123 K
0.2 × 0.1 × 0.1 mm

#### Data collection  

Agilent Xcalibur (Ruby, Gemini ultra) diffractometer
Absorption correction: analytical [*CrysAlis PRO* (Agilent, 2012 ▶), using a multi-faceted crystal model based on expressions derived by Clark & Reid (1995 ▶)] *T*_min_ = 0.162, *T*_max_ = 0.462
14167 measured reflections
1368 independent reflections
1276 reflections with *I* > 2σ(*I*)
*R*_int_ = 0.022

#### Refinement  

*R*[*F* ^2^ > 2σ(*F* ^2^)] = 0.009
*wR*(*F* ^2^) = 0.021
*S* = 1.12
1368 reflections
100 parameters
All H-atom parameters refined
Δρ_max_ = 0.44 e Å^−3^
Δρ_min_ = −0.29 e Å^−3^

### 

Data collection: *CrysAlis PRO* (Agilent, 2012 ▶); cell refinement: *CrysAlis PRO*; data reduction: *CrysAlis PRO*; program(s) used to solve structure: *OLEX2.solve* (Bourhis *et al.*, 2014 ▶); program(s) used to refine structure: *SHELXL97* (Sheldrick, 2008 ▶); molecular graphics: *DIAMOND* (Brandenburg & Putz, 2012 ▶); software used to prepare material for publication: *OLEX2* (Dolomanov *et al.*, 2009 ▶).

## Supplementary Material

Crystal structure: contains datablock(s) I. DOI: 10.1107/S1600536814012343/pk2522sup1.cif

Structure factors: contains datablock(s) I. DOI: 10.1107/S1600536814012343/pk2522Isup2.hkl

Supporting information file. DOI: 10.1107/S1600536814012343/pk2522Isup3.mol

CCDC reference: 1005538

Additional supporting information:  crystallographic information; 3D view; checkCIF report

## Figures and Tables

**Table 1:** Hydrogen-bond geometry (Å, °)

*D*—H⋯*A*	*D*—H	H⋯*A*	*D*⋯*A*	*D*—H⋯*A*
N2—H2*A*⋯Cl1	0.85 (2)	2.62 (2)	3.4437 (16)	162.8 (19)
N2—H2*B*⋯Cl1^i^	0.85 (2)	2.52 (2)	3.3594 (17)	169.0 (19)
N1—H1*A*⋯Cl1^i^	0.91 (2)	2.42 (2)	3.3155 (17)	170.2 (17)
N4—H4*A*⋯Cl1^ii^	0.89 (3)	2.81 (3)	3.6330 (19)	154 (2)
N1—H1*B*⋯Cl1^ii^	0.90 (3)	2.45 (3)	3.3440 (17)	173 (2)
N2—H2*C*⋯N4^iii^	0.86 (2)	2.16 (2)	3.020 (2)	178 (2)
N3—H3*A*⋯Cl1	0.84 (3)	2.75 (3)	3.583 (2)	171 (2)
N4—H4*B*⋯Cl1	0.93 (4)	2.64 (4)	3.544 (2)	166 (3)
N4—H4*C*⋯Cl1^iv^	0.82 (5)	2.82 (5)	3.608 (2)	163 (4)
N1—H1*C*⋯N3^v^	0.89 (3)	2.08 (3)	2.970 (2)	178.1 (19)
N3—H3*B*⋯Cl1^vi^	0.83 (3)	2.80 (3)	3.616 (2)	168 (3)

Symmetry codes: (i) 
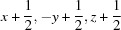
; (ii) 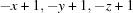; (iii) 
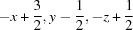
; (iv) 
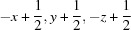
; (v) 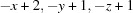; (vi) 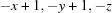.

**Fig. 1.**
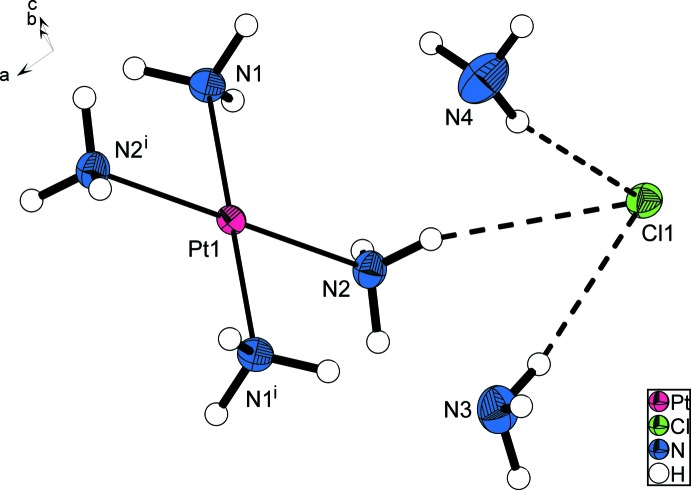
: Crystal structure of the title compound with labeling and displacement ellipsoids drawn at the 50% probability level. Symmetry code: (i) 2 - x, 1 - y, 1 - z.

**Fig. 2.**
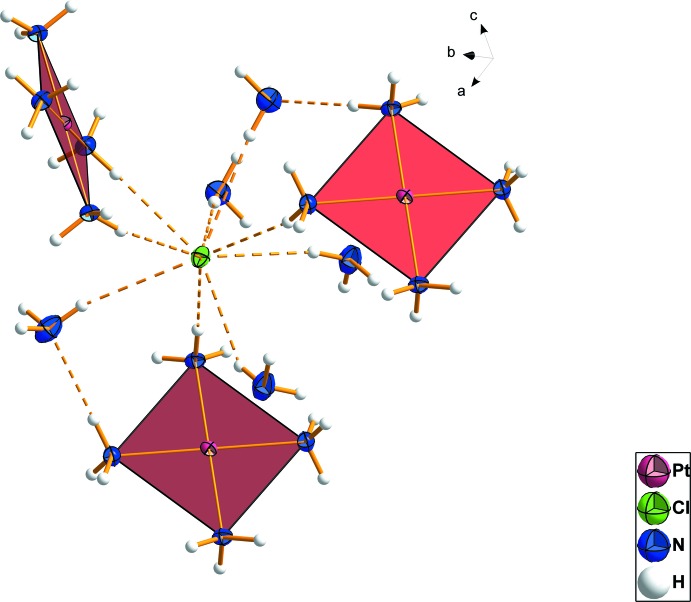
: The chloride anion is shown with its surrounding molecules. The predominant bond type is hydrogen bonding. Displacement ellipsoids are drawn at the 50% probability level.

**Fig. 3.**
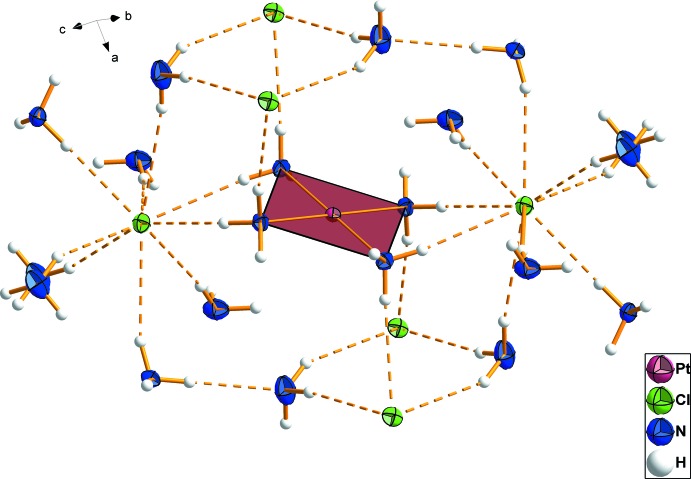
: Extended network of hydrogen bonds in the crystal structure. The solvent ammonia molecules are oriented to optimize the hydrogen bond geometry. Displacement ellipsoids are drawn at the 50% probability level.

## supplementary crystallographic information

### S1. Comment

The crystal structure of the title compound was determined in the course of investigations regarding the reactivity of carbohydrates towards metal cations in liquid ammonia.

In the crystal structure the platinum cation forms a homoleptic ammine complex with a square-planar coordination geometry. The Pt—N bond lengths are 2.0471 (16) Å and 2.0519 (15) Å, respectively. This is in good accordance with the bond lengths given by Smolentsev *et al.* (2010). The angles N—Pt—N are 89.24 (6)° and 90.76 (6)°, and within the complex, ammonia ligands opposite to each other have staggered hydrogen atom positions (Fig 1).

The chloride anion shows nine direct contacts to hydrogen atoms of ammonia molecules either bound in the complex or to solvate molecules, forming a network of hydrogen bonds (Fig. 2 and Fig. 3). The N—H···Cl bond angles range between 154 (2)° and 173 (2)° whereas N—H···Cl bond lengths have values between 2.42 (2) Å and 2.82 (5) Å. The two occurring N—H···N bridges are nearly linear, with bond angles of 178 (2)° and 178.1 (19)° and bond lengths considerably less than the sum of the van der Waals radii of nitrogen and hydrogen [2.16 (2) Å and 2.08 (3) Å]. This gives strong evidence that the arrangement of the overall structure is significantly driven by the energy contribution of N—H···N and N—H···Cl hydrogen bonds.

### S2. Experimental

0.25 g (1.0 mmol) PtCl_2_ and 0.21 g (1.00 mmol) *N*-acetylglucosamine were placed under argon atmosphere in a reaction vessel and 40 ml of dry liquid ammonia were condensed. The mixture was stored at 237 K for one week to ensure that all substances were completely dissolved. The flask was then stored at 161 K for five months. After that period, clear colorless crystals of the title compound were found at the bottom of the flask.

### S3. Refinement

The crystal structure does not show any features where special refinement methods have to be applied. All hydrogen atoms could be located in difference map and both bond angle/bond length and isotropic displacement parameters were refined.

### Crystal data

**Table:**

[Pt(NH_3_)_4_]Cl_2_·4NH_3_	*F*(000) = 384
*M**_r_* = 402.25	*D*_x_ = 1.991 Mg m^−^^3^
Monoclinic, *P*2_1_/*n*	Mo *K*α radiation, λ = 0.71073 Å
*a* = 7.6641 (2) Å	Cell parameters from 9522 reflections
*b* = 10.1601 (3) Å	θ = 3.1–30.6°
*c* = 8.7797 (2) Å	µ = 10.83 mm^−^^1^
β = 100.975 (3)°	*T* = 123 K
*V* = 671.15 (3) Å^3^	Block, clear light colourless
*Z* = 2	0.2 × 0.1 × 0.1 mm

### Data collection

**Table:**

Agilent Xcalibur (Ruby, Gemini ultra) diffractometer	1368 independent reflections
Radiation source: fine-focus sealed tube	1276 reflections with *I* > 2σ(*I*)
Graphite monochromator	*R*_int_ = 0.022
phi and ω scans	θ_max_ = 26.4°, θ_min_ = 3.1°
Absorption correction: analytical [*CrysAlis PRO* (Agilent, 2012), using a multi-faceted crystal model based on expressions derived by Clark & Reid (1995)]	*h* = −9→9
*T*_min_ = 0.162, *T*_max_ = 0.462	*k* = −12→12
14167 measured reflections	*l* = −10→10

### Refinement

**Table:**

Refinement on *F*^2^	Primary atom site location: iterative
Least-squares matrix: full	Secondary atom site location: difference Fourier map
*R*[*F*^2^ > 2σ(*F*^2^)] = 0.009	Hydrogen site location: inferred from neighbouring sites
*wR*(*F*^2^) = 0.021	All H-atom parameters refined
*S* = 1.12	*w* = 1/[σ^2^(*F*_o_^2^) + (0.0076*P*)^2^ + 0.3355*P*] where *P* = (*F*_o_^2^ + 2*F*_c_^2^)/3
1368 reflections	(Δ/σ)_max_ = 0.001
100 parameters	Δρ_max_ = 0.44 e Å^−^^3^
0 restraints	Δρ_min_ = −0.29 e Å^−^^3^

### Special details

**Table:**

Experimental. Absorption correction: CrysAlisPro, Agilent Technologies, Analytical numeric absorption correction using a multifaceted crystal model based on expressions derived by R.C. Clark & J.S. Reid. (Clark & Reid, 1995)
Geometry. All e.s.d.'s (except the e.s.d. in the dihedral angle between two l.s. planes) are estimated using the full covariance matrix. The cell e.s.d.'s are taken into account individually in the estimation of e.s.d.'s in distances, angles and torsion angles; correlations between e.s.d.'s in cell parameters are only used when they are defined by crystal symmetry. An approximate (isotropic) treatment of cell e.s.d.'s is used for estimating e.s.d.'s involving l.s. planes.
Refinement. Refinement of *F*^2^ against ALL reflections. The weighted *R*-factor *wR* and goodness of fit *S* are based on *F*^2^, conventional *R*-factors *R* are based on *F*, with *F* set to zero for negative *F*^2^. The threshold expression of *F*^2^ > σ(*F*^2^) is used only for calculating *R*-factors(gt) *etc*. and is not relevant to the choice of reflections for refinement. *R*-factors based on *F*^2^ are statistically about twice as large as those based on *F*, and *R*- factors based on ALL data will be even larger.

### Fractional atomic coordinates and isotropic or equivalent isotropic displacement parameters (Å^2^)

**Table:**

	*x*	*y*	*z*	*U*_iso_*/*U*_eq_	
Pt1	1.0000	0.5000	0.5000	0.01239 (4)	
Cl1	0.40589 (5)	0.33782 (4)	0.26730 (5)	0.01904 (8)	
N1	0.9576 (2)	0.48294 (16)	0.72243 (19)	0.0173 (3)	
N2	0.8502 (2)	0.33479 (16)	0.43520 (18)	0.0178 (3)	
N3	0.7320 (2)	0.4290 (2)	0.0434 (2)	0.0277 (4)	
N4	0.5205 (3)	0.6754 (2)	0.3118 (2)	0.0328 (4)	
H2A	0.740 (3)	0.352 (2)	0.405 (2)	0.030 (6)*	
H2B	0.857 (3)	0.282 (2)	0.511 (3)	0.028 (6)*	
H1A	0.941 (3)	0.398 (2)	0.747 (2)	0.024 (5)*	
H4A	0.577 (4)	0.675 (3)	0.410 (4)	0.073 (10)*	
H1B	0.858 (3)	0.526 (2)	0.732 (3)	0.034 (6)*	
H2C	0.887 (3)	0.291 (2)	0.364 (3)	0.028 (6)*	
H3A	0.648 (3)	0.406 (3)	0.086 (3)	0.043 (7)*	
H4B	0.509 (4)	0.586 (4)	0.293 (4)	0.078 (10)*	
H4C	0.428 (6)	0.714 (5)	0.315 (5)	0.140 (19)*	
H1C	1.050 (4)	0.5111 (19)	0.792 (3)	0.029 (6)*	
H3B	0.688 (4)	0.486 (2)	−0.021 (4)	0.049 (9)*	
H3C	0.752 (3)	0.360 (3)	−0.014 (3)	0.058 (8)*	

### Atomic displacement parameters (Å^2^)

**Table:**

	*U*^11^	*U*^22^	*U*^33^	*U*^12^	*U*^13^	*U*^23^
Pt1	0.01155 (5)	0.01255 (5)	0.01316 (6)	0.00056 (3)	0.00256 (3)	0.00081 (3)
Cl1	0.01979 (18)	0.01656 (19)	0.0203 (2)	0.00148 (17)	0.00270 (15)	−0.00201 (17)
N1	0.0185 (8)	0.0176 (8)	0.0165 (8)	0.0000 (6)	0.0049 (7)	0.0015 (6)
N2	0.0181 (8)	0.0173 (8)	0.0177 (8)	−0.0025 (6)	0.0026 (6)	0.0012 (7)
N3	0.0256 (9)	0.0332 (10)	0.0227 (9)	0.0001 (8)	0.0003 (7)	0.0057 (8)
N4	0.0483 (11)	0.0238 (10)	0.0252 (10)	−0.0071 (9)	0.0041 (8)	0.0005 (8)

### Geometric parameters (Å, º)

**Table:**

Pt1—N1	2.0471 (16)	N2—H2B	0.85 (2)
Pt1—N1^i^	2.0471 (16)	N2—H2C	0.86 (2)
Pt1—N2^i^	2.0519 (15)	N3—H3A	0.84 (3)
Pt1—N2	2.0519 (15)	N3—H3B	0.83 (3)
N1—H1A	0.91 (2)	N3—H3C	0.90 (3)
N1—H1B	0.90 (3)	N4—H4A	0.89 (3)
N1—H1C	0.89 (3)	N4—H4B	0.93 (4)
N2—H2A	0.85 (2)	N4—H4C	0.82 (5)
			
N1^i^—Pt1—N1	179.999 (15)	Pt1—N2—H2A	112.9 (14)
N1—Pt1—N2^i^	89.24 (6)	Pt1—N2—H2B	110.5 (14)
N1^i^—Pt1—N2^i^	90.76 (6)	Pt1—N2—H2C	112.5 (14)
N1^i^—Pt1—N2	89.24 (6)	H2A—N2—H2B	106.4 (19)
N1—Pt1—N2	90.76 (6)	H2A—N2—H2C	108.9 (19)
N2—Pt1—N2^i^	180.00 (7)	H2B—N2—H2C	105 (2)
Pt1—N1—H1A	111.1 (13)	H3A—N3—H3B	105 (3)
Pt1—N1—H1B	109.9 (16)	H3A—N3—H3C	105 (2)
Pt1—N1—H1C	111.9 (16)	H3B—N3—H3C	105 (2)
H1A—N1—H1B	106.9 (19)	H4A—N4—H4B	100 (3)
H1A—N1—H1C	105.9 (18)	H4A—N4—H4C	103 (3)
H1B—N1—H1C	111 (2)	H4B—N4—H4C	115 (4)

Symmetry code: (i) −*x*+2, −*y*+1, −*z*+1.

### Hydrogen-bond geometry (Å, º)

**Table:**

*D*—H···*A*	*D*—H	H···*A*	*D*···*A*	*D*—H···*A*
N2—H2*A*···Cl1	0.85 (2)	2.62 (2)	3.4437 (16)	162.8 (19)
N2—H2*B*···Cl1^ii^	0.85 (2)	2.52 (2)	3.3594 (17)	169.0 (19)
N1—H1*A*···Cl1^ii^	0.91 (2)	2.42 (2)	3.3155 (17)	170.2 (17)
N4—H4*A*···Cl1^iii^	0.89 (3)	2.81 (3)	3.6330 (19)	154 (2)
N1—H1*B*···Cl1^iii^	0.90 (3)	2.45 (3)	3.3440 (17)	173 (2)
N2—H2*C*···N4^iv^	0.86 (2)	2.16 (2)	3.020 (2)	178 (2)
N3—H3*A*···Cl1	0.84 (3)	2.75 (3)	3.583 (2)	171 (2)
N4—H4*B*···Cl1	0.93 (4)	2.64 (4)	3.544 (2)	166 (3)
N4—H4*C*···Cl1^v^	0.82 (5)	2.82 (5)	3.608 (2)	163 (4)
N1—H1*C*···N3^i^	0.89 (3)	2.08 (3)	2.970 (2)	178.1 (19)
N3—H3*B*···Cl1^vi^	0.83 (3)	2.80 (3)	3.616 (2)	168 (3)

Symmetry codes: (i) −*x*+2, −*y*+1, −*z*+1; (ii) *x*+1/2, −*y*+1/2, *z*+1/2; (iii) −*x*+1, −*y*+1, −*z*+1; (iv) −*x*+3/2, *y*−1/2, −*z*+1/2; (v) −*x*+1/2, *y*+1/2, −*z*+1/2; (vi) −*x*+1, −*y*+1, −*z*.
